# Supplementary material for: OpenMM 7: Rapid development of high performance algorithms for molecular dynamics
Source: PLoS Comput Biol. 2017 Jul 26;13(7):e1005659. doi: 10.1371/journal.pcbi.1005659 (PMC5549999; doi:10.1371/journal.pcbi.1005659)
Supplement: S1 Source Code — (ZIP) [file pcbi.1005659.s002.zip › OpenMM-7.0.1-Source/docs/api-c++/generated/OpenMM.VerletIntegrator.html]

VerletIntegrator — OpenMM @OPENMM\_MAJOR\_VERSION@.@OPENMM\_MINOR\_VERSION@ documentation


# VerletIntegrator¶

*class* `OpenMM::``VerletIntegrator`¶
:   This is an `Integrator` which simulates a `System` using the leap-frog Verlet algorithm.

    Methods

    |  |  |
    | --- | --- |
    | `VerletIntegrator` | Create a `VerletIntegrator`. |
    | `step` | Advance a simulation through time by taking a series of time steps. |

    `VerletIntegrator`(double *stepSize*)¶
    :   Create a `VerletIntegrator`.

        |  |  |
        | --- | --- |
        | Parameters: |  |

        - **stepSize** – the step size with which to integrate the system (in picoseconds)

    void `step`(int *steps*)¶
    :   Advance a simulation through time by taking a series of time steps.

        |  |  |
        | --- | --- |
        | Parameters: |  |

        - **steps** – the number of time steps to take

High performance molecular simulation on GPUs


### Navigation

- Core Objects
- Forces
- Integrators
- Extras

©2015, Stanford University and the Authors.
|
Powered by Sphinx 1.3.1
& Alabaster 0.7.7
|
Page source
